# M_3_ Subtype of Muscarinic Acetylcholine Receptor Promotes Cardioprotection via the Suppression of miR-376b-5p

**DOI:** 10.1371/journal.pone.0032571

**Published:** 2012-03-02

**Authors:** Zhenyu Pan, Yueping Guo, Hanping Qi, Kai Fan, Shu Wang, Hua Zhao, Yuhua Fan, Jing Xie, Feng Guo, Yunlong Hou, Ning Wang, Rong Huo, Yong Zhang, Yan Liu, Zhimin Du

**Affiliations:** 1 Department of Pharmacology, the State-Province Key Laboratories of Biomedicine-Pharmaceutics of China, Key Laboratory of Cardiovascular Research, Ministry of Education, Harbin Medical University, Harbin, Heilongjiang, People′s Republic of China; 2 Department of Anesthesiology of the Second Affiliated Hospital, Harbin Medical University, Harbin, Heilongjiang, People′s Republic of China; 3 Institute of Clinical Pharmacology of the Second Affiliated Hospital, Harbin Medical University, Harbin, Heilongjiang, People′s Republic of China; University of Oldenburg, Germany

## Abstract

The M_3_ subtype of muscarinic acetylcholine receptors (M_3_-mAChR) plays a protective role in myocardial ischemia and microRNAs (miRNAs) participate in many cardiac pathophysiological processes, including ischemia-induced cardiac injury. However, the role of miRNAs in M_3_-mAChR mediated cardioprotection remains unexplored. The present study was designed to identify miRNAs that are involved in cardioprotective effects of M_3_-mAChR against myocardial ischemia and elucidate the underlying mechanisms. We established rat model of myocardial ischemia and performed miRNA microarray analysis to identify miRNAs involved in the cardioprotection of M_3_-mAChR. In H9c2 cells, the viability, intracellular free Ca^2+^ concentration ([Ca^2+^]i), intracellular reactive oxygen species (ROS), miR-376b-5p expression level, brain derived neurophic factor (BDNF) and nuclear factor kappa-B (NF-κB) levels were measured. Our results demonstrated that M_3_-mAChR protected myocardial ischemia injury. Microarray analysis and qRT-PCR revealed that miR-376b-5p was significantly up-regulated in ischemic heart tissue and the M_3_-mAChRs agonist choline reversed its up-regulation. In vitro, miR-376b-5p promoted H_2_O_2_-induced H9c2 cell injuries measured by cells viability, [Ca^2+^]i and ROS. Western blot and luciferase assay identified BDNF as a direct target of miR-376b-5p. M_3_-mAChR activated NF-κB and thereby inhibited miR-376b-5p expression. Our data show that a novel M_3_-mAChR/NF-κB/miR-376b-5p/BDNF axis plays an important role in modulating cardioprotection. MiR-376b-5p promotes myocardial ischemia injury possibly by inhibiting BDNF expression and M_3_-mAChR provides cardioprotection at least partially mediated by the downregulation of miR-376b-5p through NF-κB. These findings provide new insight into the potential mechanism by which M_3_-mAChR provides cardioprotection against myocardial ischemia injury.

## Introduction

Ischemic heart disease is the leading cause of death in the industrialized world [1]. Myocardial ischemia could induce unstable angina, arrhythmia, myocardial infarction or its neopathy, leading to sudden cardiac death [2]–[4]. While many drugs failed to efficaciously treat cardiac death with myocardial ischemia and myocardial infarction [5], recent studies have shown that M_3_ subtype of muscarinic acetylcholine receptors (M_3_-mAChR) in the heart have cardioprotective effect against myocardial ischemia [6]–[10].

MicroRNAs (miRNAs) emerge as important regulator of gene expression and have been implicated in numerous cardiovascular pathological processes, including cardiac fibrosis, cardiac hypertrophy, heart failure and cardiac arrhythmia [11]–[14]. Specially, several miRNAs including miRNA-320 [15], miRNA-21 [16] and miRNA-494 [17] are involved in myocardial ischemia. However, the potential role of miRNAs in cardioprotection of M_3_-mAChR remains to be elucidated. Therefore, the present study aimed to explore the contribution of miRNAs to cardioprotective effects mediated by M_3_-mAChR against myocardial ischemia. Our results revealed for the first time that miR-376b-5p increased myocardial ischemia injury and was involved in M_3_-mAChR's cardioprotection.

## Materials and Methods

### Ethics Statement

All experimental protocols were pre-approved by the Experimental Animal Ethic Committee of Harbin Medical University, China (Animal Experimental Ethical Inspection Protocol No. 2009104). Use of animals was confirmed with the Guide for the Care and Use of Laboratory Animals published by the US National Institutes of Health (NIH Publication No. 85–23, revised 1996).

### Reagents

Dulbecco's modified Eagle's medium (DMEM), fetal bovine serum (FBS), trypsin and other tissue culture reagents were obtained from Life Technologies, Inc (Carlsbad, CA, USA). The bicinchoninic acid (BCA) protein assay reagents were obtained from Pierce (Rockford, IL, USA). Sodium pentobarbital was obtained from Shanghai Chemicals (Shanghai, China). Choline chloride (choline, agonist of M_3_-mAChR), 4-diphenylacetoxy-N-methylpiperidine methiodide (4DAMP, antagonist of M_3_-mAChR) and lipopolysaccharide (LPS, an agonist of NF-κB) were obtained from Sigma (St. Louis, MO, USA). MiRNAs were from Shanghai GenePharma Co.,Ltd.(Shanghai, China). Lipofectamine 2000 and Alexa Fluor® 800 goat anti-mouse IgG or anti-rabbit IgG were purchased from Invitrogen (Carlsbad, CA,USA). Total RNA Purification Kit was from Norgen Biotek (Thorold, ON, Canada). The mirVanaTM qRT-PCR miRNA Detection Kit was from Ambion (Austin, TX, USA). Bio-Rad Protein Assay Kit was obtained from Bio-Rad, (Mississauga, ON, Canada). 3-(4,5-dimethylthiazol-2-yl)-2,5-diphenyltetrazolium bromide (MTT) was from Roche Diagnostics GmBH (Mannhein, Germany). 2′,7′- dichlorodihydrofluorescein diacetate (DCFH-DA) was from Molecular Probes (Eugene, USA). Anti-nuclear factor kappa-B (NF-κB) antibody and anti-GAPDH were purchased from Santa Cruz Biotechnology (Santa Cruz, CA, USA). Anti-brain derived neurophic factor (BDNF) antibody was obtained from abcam (Lake Placid, NY, USA). Luciferase reporter assay kit was from Promega (Madison, WI, USA). All other chemicals were purchased from either Sigma. The purity of all reagents was at least analytical grade.

### Establishment of rat myocardial ischemia model

Male Wistar rats (weight 280–300 g) were randomly divided into four groups: control, ischemia, choline (agonist of M_3_-mAChR), and choline+4DAMP (antagonist of M_3_-mAChR) groups. All treatments were administered via the lingual vein with doses of choline and 4DAMP as described previously [7]. Choline (10 mg/kg, i.v.) was administered 10 min before the occlusion. For choline+4DAMP group, 4DAMP (0.12 µg/kg) was administered 5 min before choline. Rat model of myocardial ischemia was established as previously described [8]. Briefly, the rats were anesthetized intraperitoneal injection with sodium pentobarbital (40 mg/kg) and the respiration of rats was controlled by the volume-controlled rodent ventilator. The ventilator setting and the level of anesthesia were adjusted to maintain the animal in an anaesthetize status without spontaneous breathing efforts. A left thoracotomy was performed at the 3rd–4th rib and a segment of saline-soaked 5-0 sutures was looped around the left anterior descending (LAD) coronary artery, near its origin from the left coronary artery. Successful occlusion was observed by electrocardiographic ST-segment elevation. The chest was closed before the rats were weaned from the ventilator and extubated. Rats were put into cage to breed with normal water and feeds after waking up. Mortality rate of the rats were calculated and compared after 24 h myocardial infarction. After 24 h ischemia, rats were reanesthetized and the hearts were removed for the measurement of myocardial infarct size and microRNA microarray analysis. Control group without drugs was handled in the same manner except that the coronary artery was not ligated.

### Measurement of myocardial infarct size

Ventricular tissues were dissected from the animals after 24 h ischemia and kept overnight at −4°. Frozen ventricles were sliced into 2 mm thick sections, and then incubated in 1% triphenyltetrazolium chloride (TTC) at 37°C in 0.2 M Tris buffer (pH 7.4) for 30 min. The non-ischemia area was stained as red and infarct area grey white. Size of the infarcted area was estimated by weight as a percentage of the left ventricle [7].

### MicroRNA microarray analysis

After 24 h ischemia, surrounding tissues of infarcted area from ventricular were departed for microRNA microarray analysis by using hybridization to *μ*Paraflo®microfluidics microarrays (LC Sciences) in the version of the Sanger miRBase database Release 10.1 (Release 12, http://microrna.sanger.ac.uk/). Each detection probe consisted of a chemically modified nucleotide-coding segment complementary to the target miRNA or other target and a spacer segment of polyethylene glycol to extend the coding segment away from the substrate. Each miRNA probe was represented 5 times on a single microarray, and the control probes were spiked into the RNA samples before the labeling. The detection probes were prepared by *in situ* synthesis using PGR (photogenerated) chemistry to allow highly sensitive and specific detection of miRNAs. The *μ*Paraflo® technology enabled on-chip synthesis, ensuring high probe quality and tight process control.

### Cell culture

H9c2 myoblast cell line (the rat embryonic ventricular myocardial cell line) were cultured and maintained as monolayer in high glucose DMEM, supplemented with 10% heat inactivated FBS, 100 U/ml penicillin and 100 µg/ml streptomycin, at 37°C in a humidified incubator with 5% CO_2_. H9c2 cells were plated at a density of 5000 cells/cm^2^ and allowed to proliferate in growth medium. Medium was changed every 3 days.

### Transfection of miRNAs and miRNA inhibitor

Rat miR-376b-5p, miR-539, 2′-O-methyl-modified antisense oligoribonucleotides of miR-376b-5p (AMO-376b-5p, miR-376b-5p inhibitor) and miR-376b-5p negative control (miR-NC) were synthesized by Shanghai GenePharma. The sequence of AMO-376b-5p was antisense of the mature miRNA sequence (for rat: 5′-GUGGAUAUUCCUUCUAUGGUUA-3′) and miR-NC was a random sequence. AMO-376b-5p contained 2′-O-methyl modification at every base and a 3′C3-containing amino linker.

H9c2 cells were transfected with miR-NC (100 nM), miR-376b-5p (100 nM), miR-539 (100 nM), miR-376b-5p (100 nM) and AMO-376b-5p (100 nM) using lipofectamine 2000 according to the manufacturer's instruction and incubated at 37°C for 24 h. Subsequently, cells were maintained in the culture until analysis [11].

### MTT assay

H9c2 cells were seeded into 96-well plates at the density of 2×10^4^/well. After 24 h, MiR-539, miR-NC, miR-376b-5p and AMO-376b-5p were transfected and incubated for 24 h, followed by treatment with H_2_O_2_ (50 µM) for 12 h. Then 10 µl MTT reagents (0.5 mg/ml) was added and incubated for 4 h. After dissolving the formazine granulars with 150 µl dimethyl sulphoxide (DMSO), the absorbance at 570 nm was measured by using a microplate reader. The cell viability = (A_control group_−A_experimental group_)/A_control group_×100%.

### Measurement of intracellular free Ca^2+^

H9c2 cells were seeded into 6-well plates. The cells were treated with H_2_O_2_ (50 µM) for 12 h before transfection with miRNAs. The [Ca^2+^]i levels were measured 24 h after transfection. Pluronic F-127 was used as a dispersing agent to facilitate the loading of cells with a final concentration of 0.012% Fluo-3/AM in the loading medium. Changes in the fluorescence intensity of Fluo-3/AM-loaded cells were detected by laser scanning confocal microscopy (FV-300; Olympus, Tokyo, Japan) at 488 nm excitation and 530 nm emission wavelengths. The acquisition rate was one frame at 10 s intervals and [Ca^2+^]_i_ was monitored for 300 s.

### Measurement of intracellular ROS

Intracellular reactive oxygen species (ROS) were stained using DCFH-DA. H9c2 cells were seeded into 6-well plates. The cells were treated with H_2_O_2_ (50 µM) for 12 h before transfection with miRNAs. The intracellular ROS levels were measured 24 h after transfection. The cells were washed with serum-free DMEM and incubated with 10 µmol/L DCFH-DA in the loading medium in 5% CO_2_/95% air at 37°C for 20 min. After DCFH-DA was removed, the cells were washed twice and the DCF fluorescence intensity was observed and photographed by laser scanning confocal microscopy (FV-300; Olympus, Tokyo, Japan) at 488 nm excitation and 525 nm emission wavelengths. The acquisition rate was one frame at 10 s intervals and ROS was monitored for 300 s.

### RT-PCR

Total RNA was isolated from H9c2 cells or rat myocardium with Total RNA Purification Kit. qRT-PCR for miR-376b-5p, miR-539 was performed on a GeneAmp 5700 thermocycler using mirVanaTM miRNA Detection Kit following the manufacturer's instructions. U6 was used as an internal control. The primers were as follows: U_6_ primers q5′-CTCCGATAGATCTGCCCTCTTGAA-3′ (forward), 5′-CGCTTCACGAATTTGCGTGTCAT-3′ (reverse). miR-376b-5p primers 5′-GGGGGTGGATATTCCTTCT-3′ (forward), 5′-CAGTGCGTGTCGTGGAGT-3′ (reverse). miR-539 primers 5′-GGGGGAGAAATTATCCTT-3′ (forward), 5′-TGCGTGTCGTGGAGTC-3′ (reverse).

### Western blot analysis

Whole cell extract and nuclear extract were extracted from H9c2 cells. The protein content was determined with Bio-Rad Protein Assay Kit using bovine serum albumin as the standard. Protein sample (50 µg) was fractionated by 7.5–10% SDS-PAGE and transferred to nitrocellulose membranes. The membranes were blocked in 5% milk PBS-Tween20 (PBST) for 3 h and incubated overnight at 4°C with the following primary antibodies: BDNF, NF-κB or GAPDH as internal controls, followed by incubation with Alexa Fluor® 800 goat anti-mouse IgG or anti-rabbit IgG (1∶10000) for 1 h. The images were captured on the Odyssey Infrared Imaging System (LI-COR Bioscience, Lincoln, NE, USA) and band intensity was quantified using Odyssey v1.2 software.

### Luciferase activity assay

A fragment of the 3′-untranslated region (3′-UTR) of BDNF containing the putative miR-376b-5p binding sequence was cloned into psiCHECK-2 (Renilla luciferase vector) and the construct (0.5 µg) was transfected into HEK 293T cells with miR-NC, miR-376b-5p, AMO-376b-5p or AMO-376b-5p negative control (AMO-NC). After 24 h, luciferase activities were measured with a dual luciferase reporter assay kit on a luminometer (Lumat LB9507) (EG&G Berthold, Bad Wildbad, Germany).

### Statistical analysis

Average data were presented as mean ± standard error and were analyzed by analysis of variance (ANOVA) followed by Bonferroni's post-hoc test. *P*<0.05 was considered statistically significant.

## Results

### MicroRNAs are involved in M_3_-mAChR mediated cardioprotection against myocardial ischemia in rat

**Figure 1:**
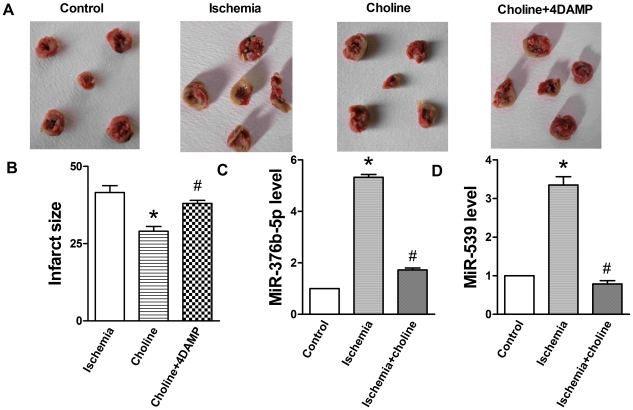
MiRNA is involved in M_3_-mAChR mediated cardioprotection against myocardial ischemia in rat. Choline (10 mg/kg, i.v.) was administrated 10 min before occlusion. 4DAMP (0.12 µg/kg) was administered 5 min before choline. (A) Representative infarct photographs. (B) Infarct size. Values were expressed as mean ± SEM; n = 8; *P<0.05 vs. Control; ^#^ *P*<0.05 vs. Choline. (C) Choline reversed ischemia-induced miR-376b-5p up-regulation. (D) Choline reversed ischemia-induced miR-539 up-regulation. Values were expressed as mean ± SEM; n = 3; *P<0.05 vs. Control, ^#^ *P*<0.05 vs. Ischemia.

We established rat model of myocardial ischemia and examined mortality rate and myocardial infarct size. Choline significantly reduced the infarct size, which was reversed by 4DAMP (Figure 1A and B). As expected, choline group showed an obvious decrease in mortality rate (8.33%, *n* = 12, 1 died) compared to control group subjected to the same procedure (28.6%, *n* = 14, 4 died), and 4DAMP increased mortality rate (14.3%, *n* = 14, 2 died) compared to choline group. Taken together, these results confirmed that M_3_-mAChR provides cardioprotection against myocardial ischemia.

To determine whether miRNAs are involved in M_3_-mAChR mediated cardioprotection, we performed miRNA microarray analysis and identified 12 differentially upregulated miRNAs upon myocardial ischemia, among which 10 miRNAs were differentially downregulated by M_3_-mAChR agonist choline (Table 1). While miR-199a, miR-376b-5p, miR-539 and miR-106 were all significantly upregulated (more than 3 times compared to the control), only miR-376b-5p and miR-539 were downregulated to the level similar to the control group after treatment with choline.

**Table 1:** The levels of differentially expressed miRNAs in the rat sufferred from myocardial ischemia or administrated with choline after ischemia.

Probe_ID	Control	Ischemia	Ischemia-choline
rno-miR-199a	1	12.7	2.30
rno-miR-449	1	1.80	1.20
**rno-miR-376b-5p**	1	9.70	1.87
**rno-miR-539**	1	4.00	0.81
rno-miR-106	1	3.15	0.15
rno-miR-92	1	2.52	1.11
rno-miR-135b	1	1.80	1.16
rno-miR-349	1	1.58	0.41
rno-miR-122a	1	1.65	1.10
rno-miR-18	1	2.00	1.03

Male Wistar rats were divided into control, ischemia and choline chloride (choline, agonist of M_3_-mAChR) groups. Myocardial ischemia model was induced for 24 h. Choline (10 mg/kg, i.v.) was administrated 10 min before occlusion. After 24 h ischemia, ventricular tissues were dissected for miRNA microarray analysis. MiRNAs levels were calculated based on the normalization to control group (set as 1).

To confirm the expression changes of miR-376b-5p and miR-539 in myocardial ischemia, next we performed qRT-PCR analysis and found that miR-376b-5p and miR-539 were significantly upregulated upon myocardial ischemia and down-regulated by choline (*P*<0.05, Figure 1C and D). These data demonstrate that miR-376b-5p and miR-539 are significantly upregulated in ischemic heart of the rats and M_3_-mAChR agonist choline could reverse their upregulation. Therefore, miR-376b-5p and miR-539 appear to be involved in M_3_-mAChR mediated cardioprotection against myocardial ischemia.

### MiR-376b-5p promotes H_2_O_2_ induced H9c2 cells injury

**Figure 2:**
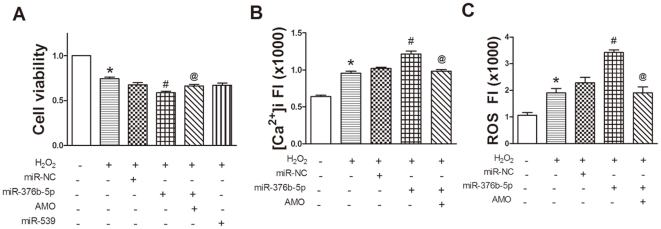
MiR-376b-5p promotes H_2_O_2_-induced H9c2 cells injury. H9c2 cells were pre-treated with H_2_O_2_ (50 µM) for 12 h and then transfected with miR-376b-5p negative control (miR-NC), miR-376b-5p, miR-539, and 2′-O-methyl-modified antisense oligoribonucleotides of miR-376b-5p (AMO-376b-5p, miR-376b-5p inhibitor) for 24 h. (A) MTT assay. MiR-376b-5p, not miR-539, reduced cell viability in H_2_O_2_-treated H9c2 cells. n = 19 values from 5 independent experiments. (B) Intracellular Ca^2+^ concentration ([Ca^2+^]i) fluorescence intensity. MiR-376b-5p significantly increased [Ca^2+^]i and this effect was reversed by AMO-376b-5p (AMO). n = 29 cells from 5 independent experiments. (C) Reactive oxygen species (ROS) fluorescence intensity. MiR-376b-5p significantly increased relative fluoresence intensity of ROS. n = 12 cells from 3 independent experiments. Values were expressed as mean ± SEM; *P<0.05 vs. Control, ^#^ *P*<0.05 vs. H_2_O_2_+miR-NC, ^@^ *P*<0.05 vs. H_2_O_2_+miR-376b-5p. FI: fluorescence intensity.

To determine the involvement of miR-376b-5p and miR-539 in myocardial ischemia, we used H9c2 cells as an *in vitro* model. MTT assay showed that miR-376b-5p significantly enhanced H_2_O_2_-induced inhibition of H9c2 cells viability (*P*<0.05), which was reversed by AMO-376b-5p. However, miR-539 had no significant effect on H_2_O_2_-induced inhibition of H9c2 cells viability (Figure 2A). These results suggest that miR-539 is unlikely involved in myocardial ischemia and we focused on miR-376b-5p in the following experiments.

The overload of intracellular free Ca^2+^ concentration ([Ca^2+^]i) is an important cause of cell injury [7], [9]. Therefore, we examined [Ca^2+^]i in H_2_O_2_-treated H9c2 cells and found that miR-376b-5p significantly increased the overload of [Ca^2+^]i (*P*<0.05) while AMO-376b-5p reversed this increase (Figure 2B).

Moreover, miR-376b-5p significantly increased the intracellular ROS in H_2_O_2_-treated H9c2 cells (*P*<0.05) and this effect was reversed by AMO-376b-5p (Figure 2C). Taken together, these results suggest that miR-376b-5p promotes H_2_O_2_-induced H9c2 cells injury.

### M_3_-mAChR inhibits H_2_O_2_ induced upregulation of miR-376b-5p in H9c2 cells

**Figure 3:**
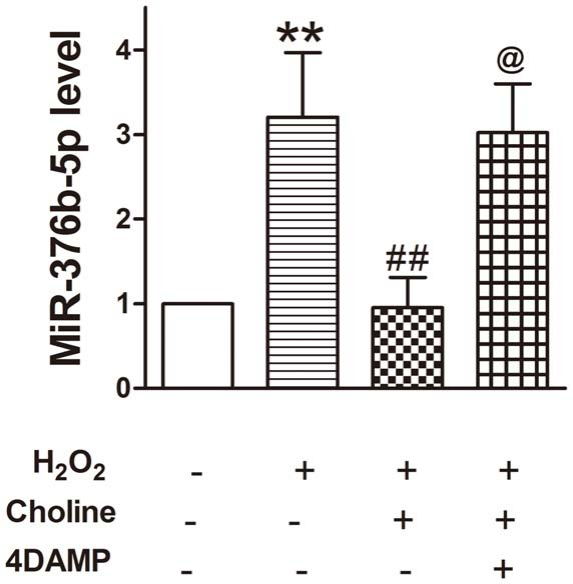
M_3_-mAChR inhibits the up-regulation of miR-376b-5p stimulated by H_2_O_2_ in H9c2 cells. H9c2 cells were co-incubated with H_2_O_2_ (50 µM), choline (1 mM) and 4DAMP (5 nM) for 12 h. MiR-376b-5p expression was determined by qRT-PCR. U6 served as an internal control for the normalization. Values were expressed as mean ± SEM; n = 4; **P<0.01 vs. Control, ^##^ *P*<0.01 vs. H_2_O_2_+choline, ^@^ *P*<0.05 vs. H_2_O_2_+choline+4DAMP.

To examine further miR-376b-5p expression in ischemic myocardial cells and the effects of M_3_-mAChR on miR-376b-5p expression in vitro, H9c2 cells were co-incubated with H_2_O_2_ (50 µM), choline (1 mM) or 4DAMP (5 nM) for 12 h. The results showed that H_2_O_2_ significantly upregulated the miR-376b-5p expression (*P*<0.01) and this effect was antagonized by M_3_-mAChR agonist choline (Figure 3). Furthermore, M_3_-mAChR antagonist 4DAMP reversed the effect of choline on miR-376b-5p. These results are consistent with the results of gene array analysis verified by qRT-PCR (Figure 1C) and suggest that M_3_-mAChR inhibits the up-regulation of miR-376b-5p stimulated by H_2_O_2_ in H9c2 cells.

### BDNF is a target gene of miR-376b-5p in cardiac myocytes

**Figure 4:**
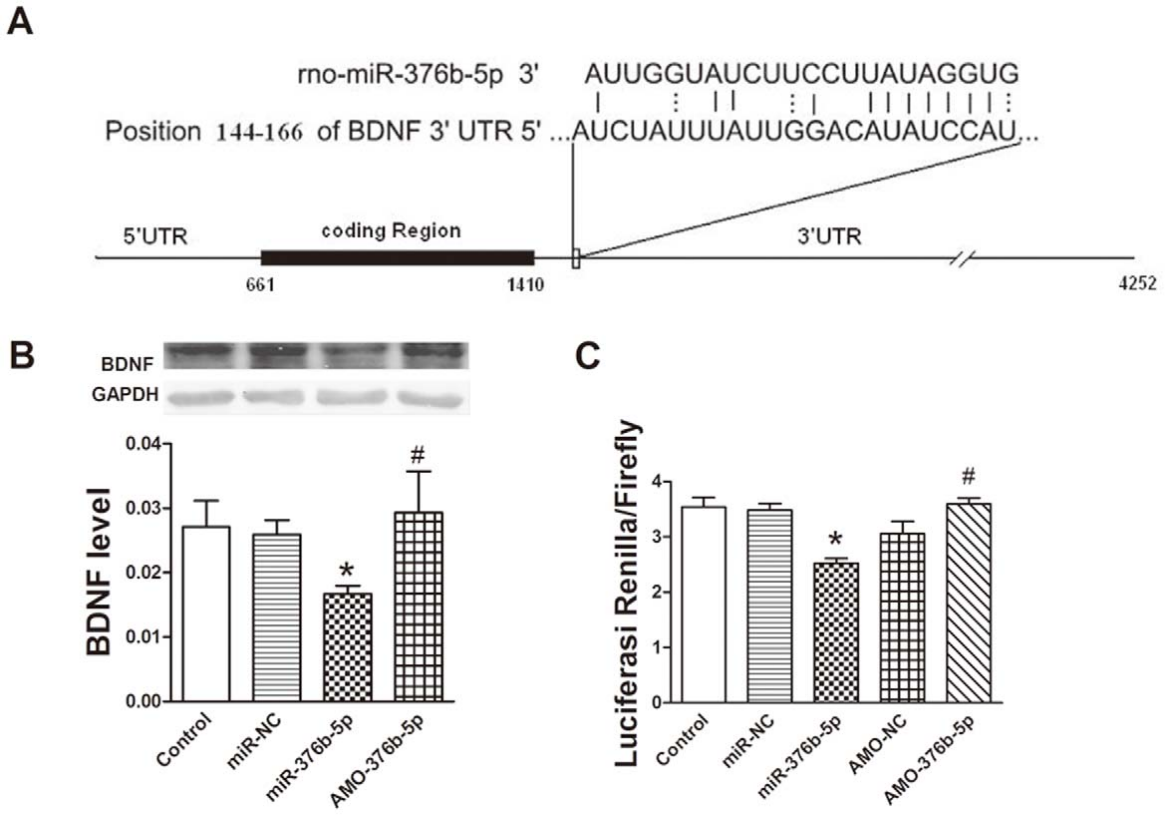
BDNF is a target gene of miR-376b-5p in cardiac myocytes. (A) MiR-376b-5p binding site in 3′-untranslated region (3′-UTR) of BDNF gene. (B) Inhibitory effects of miR-376b-5p on BDNF expression at the protein level. BDNF level was determined by Western blot after H9c2 cells were transfected with miR-376b-5p negative control (miR-NC), miR-376b-5p and 2′-O-methyl-modified antisense oligoribonucleotides of miR-376b-5p (AMO-376b-5p, miR-376b-5p inhibitor) for 24 h. GAPDH served as loading control. Top, representative Western blots; bottom, quantitation as mean ± SEM. Note: n = 3; *P<0.05 vs. miR-NC, ^#^ *P*<0.05 vs. miR-376b-5p. (C) Luciferase assay for direct inhibitory effects of miR-376b-5p on BDNF expression. The reporter construct was transfected into HEK 293T cells with miR-NC, miR-376b-5p, AMO-376b-5p or AMO-376b-5p negative control (AMO-NC). Luciferase activity was normalized by Firefly luciferase signal in HEK 293T cells. Following transfection for 24 h, luciferase activities were measured on a luminometer. Values were expressed as mean ± SEM; n = 3; *P<0.05 vs. miR-NC, ^#^ *P*<0.05 vs. AMO-NC.

To further explore the role of miR-376b-5p in myocardial ischemia, we predicted the targets of miR-376b-5p by targetScan Release 5.1 online (www.targetscan.org) and focused on BDNF (Figure 4A). Next we determined the expression of BDNF at the protein level in H9c2 cells and found that miR-376b-5p significantly reduced the expression of BDNF (*P*<0.05), and this reduction was reversed by AMO-376b-5p (Figure 4B). These results suggest that BDNF is a target gene of miR-376b-5p.

To confirm that miR-376b-5p could directly bind to BDNF gene and inhibit its expression, a reporter construct with the insertion of a fragment of the 3′-UTR of BDNF mRNA containing the predicted miR-376b-5p binding site was transfected into HEK 293T cells. Luciferase assay showed that miR-376b-5p significantly reduced the luciferase activity (*P*<0.05) while AMO-376b-5p significantly increased the luciferase activity (*P*<0.05) (Figure 4C). Taken together, these data prove that miR-376b-5p directly inhibits BDNF expression.

### NF-κB mediates the inhibitory effect of M_3_-mAChR on miR-376b-5p expression in H9c2 cells

**Figure 5:**
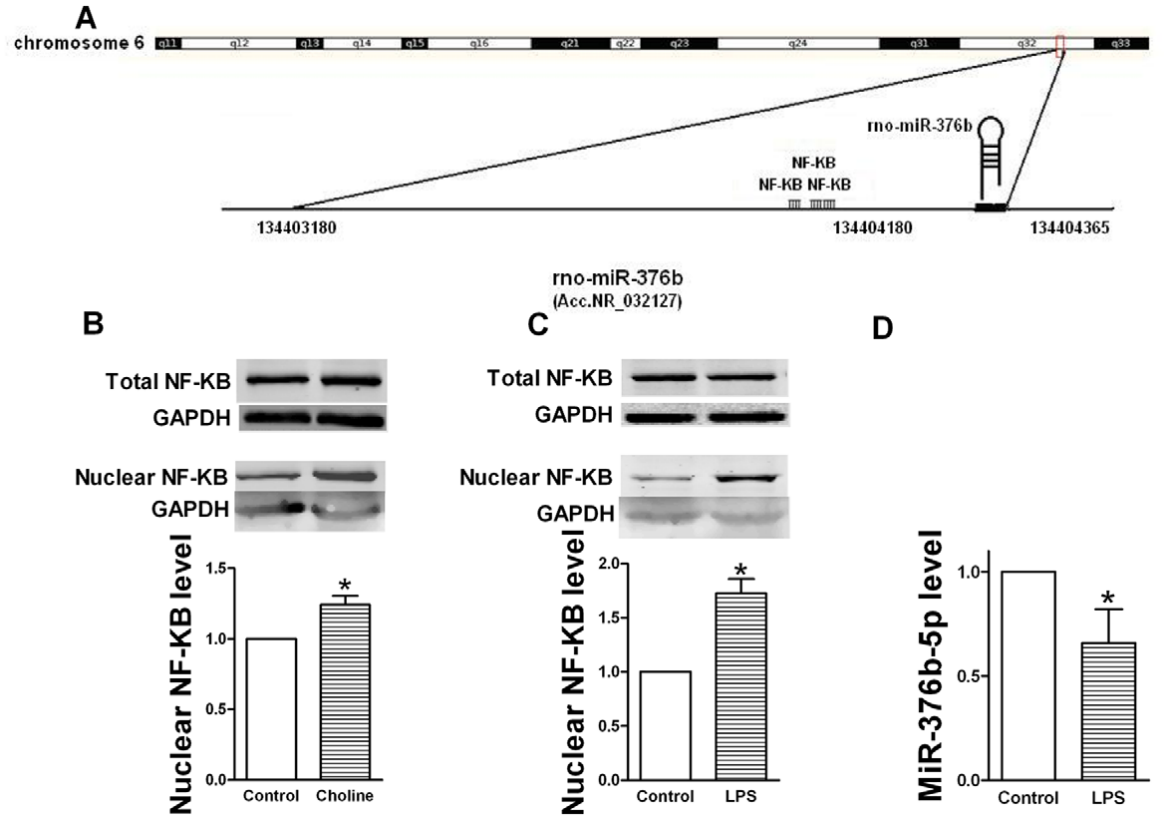
NF-κB mediates the inhibitory effect of M_3_-mAChR on miR-376b-5p expression. GAPDH as an internal control normalized NF-κB band for western blot. U6 as an internal control normalized miR-376b-5p for quantitative real-time RT-PCR (qRT-PCR). (A) Chromosomal organization of the miRNA-376b gene locus on rat chromosome 6q32 indicating three NF-κB binding sites in the pre-miR-376b gene promoter. (B) Choline activated NF-κB in H9c2 cells. Top, representative Western blots of total NF-κB; Middle, representative Western blots of nuclear NF-κB; bottom, quantitation of nuclear NF-κB as mean ±SEM Note: n = 4; *P<0.05 versus Control. (C) LPS activated NF-κB in H9c2 cells. Top, representative Western blots of total NF-κB; Middle, representative Western blots of nuclear NF-κB; bottom, quantitation of nuclear NF-κB as mean ±SEM. Note: n = 6; *P<0.05 versus Control. (D) LPS inhibited miR-376b-5p expression in H9c2 cells. MiR-376b-5p expression was determined by qRT-PCR. Values were expressed as mean ± SEM; n = 5; *P<0.05 vs. Control.

Previous studies demonstrated that M_3_-mAChR could activate NF-κB [18]–[20]. Moreover, 3 binding sites of NF-κB were predicted on the promoter of miR-376b-5p gene by TFSEARCH (Figure 5A). Therefore, we postulated that NF-κB possibly mediates the inhibitory effects of M_3_-mAChR on miR-376b-5p expression.

To verify that M_3_-mAChR could activate NF-κB, we determined nuclear NF-κB protein level in H9c2 cells. Western blot analysis showed that nuclear NF-κB level was significantly increased (*P*<0.05, Figure 5B) after treatment with choline with no significant variance of total NF-κB level, suggesting that M_3_-mAChR could activate NF-κB.

Next we measured miR-376b-5p expression by qRT-PCR in H9c2 cells treated with LPS (100 ng/ml), which is known to activate NF-κB in many previous studies [21]–[23]. As expected, LPS significantly activated NF-κB (*P*<0.05, Figure 5C) with no significant variance of total NF-κB level. Furthermore, LPS significantly reduced miR-376b-5p expression (*P*<0.05, Figure 5D). Collectively, these data indicate that NF-κB mediates the inhibitory effect of M_3_-mAChR on miR-376b-5p expression.

## Discussion

In the present study, for the first time, we found that miR-376b-5p inhibited the expression of BDNF and miR-376b-5p promoted H_2_O_2_-induced H9c2 cells injury, indicating that miR-376b-5p promoted myocardial ischemia injury possibly by inhibiting the expression of BDNF. Moreover, we found that M_3_-mAChR inhibited miR-376b-5p expression by activating NF-κB. These findings provide new insight into the potential mechanism of cardioprotection of M_3_-mAChR against myocardial ischemia.

First, we observed that M_3_-mAChR decreased infarct size and mortality rate in rat models, consistent with previous studies showing that M_3_-mAChR has the protective effect against myocardial ischemia [6]–[10]. Next, to explore the underlying mechanisms responsible for our observation, we focused on miRNAs, given their emerging role in the pathogenesis of the cardiovascular system including myocardial ischemia [11]–[17]. Based on microarray analysis and further validation by qRT-PCR, we postulated that the upregulation of miR-376b-5p and miR-539 is involved in myocardial ischemia and M_3_-mAChR may protect against myocardial ischemia via the downregulation of miR-376b-5p and miR-539 expression.

H9c2 cells were treated with H_2_O_2_ for 12 h to simulate myocardial ischemia injury, which was employed as in vitro model to test our hypothesis. In our experiment, the miRNA transfection efficiency was measured by real-time PCR. MiR-376b-5p level was upregulated more than 3 times after transfection (Figure S1), demonstrating the efficient transfection. Notably, miR-376b-5p but not miR-539 was implicated in reduced H9c2 cells viability induced by H_2_O_2_. Therefore, miR-376b-5p was chosen for further characterization.

TargetScan Release 5.1 was used to predict the target genes of miR-376b-5p. Although 1134 potential targets of miR-376b-5p were predicted, only a few of them have been reported to be involved in myocardial ischemia, including insulin-like growth factor 2 mRNA binding protein 2 [24], [25], ryanodine receptor 2 [26]–[28] and BDNF [29]–[35]. Given recent studies demonstrating that BDNF was also expressed in the heart where it protected against myocardial ischemia injury [29]–[35], we proposed that BDNF is one potential target of miR-376b-5p in the heart. Our Western blot analysis and luciferase assay experiments demonstrated that miR-376b-5p could directly bind to BDNF gene and inhibit its expression, providing support for our hypothesis that miR-376b-5p promotes myocardial ischemia injury possibly via the downregulation of BDNF.

To determine the potential mechanism by which M_3_-mAChR inhibited miR-376b-5p expression, TFSEARCH was used to identify transcription factors that bind to the promoter of miR-376b-5p gene. Interestingly, we found 3 binding sites of NF-κB on the promoter, with percentage scores as 85, 85.8 and 91.4, respectively. NF-κB has been shown to provide myocardial protection by diverse mechanisms including increase of cardiac Bcl-2 [36], [37], induction of manganese superoxide disumutase [38], and inhibition of leukocyte adhesion, cytokines, and chemokines [39]–[42]. In the preconditioning episodes, NF-κB is activated and pharmacological inhibition of NF-κB abolishes its cardioprotective effect [36], [43], [44]. Moreover, several studies have demonstrated that M_3_-mAChR activated NF-κB [18]–[20], which may be mediated by intracellular Ca^2+^ and PKC-dependent signaling pathways [19], [45]. In the present study, we demonstrated that NF-κB was activated when M_3_-mAChR was activated by M_3_-mAChR agonist choline in H9c2 cells. Furthermore, LPS, NF-κB activator, significantly reduced miR-376b-5p expression. Collectively, these data strongly suggest that M_3_-mAChR inhibits miR-376b-5p expression via the activation of NF-κB.

In conclusion, our results help establish that a novel M_3_-mAChR/NF-κB/miR-376b-5p/BDNF axis plays an important role in modulating cardioprotection. To our knowledge, this is the first report to demonstrate that miR-376b-5p promotes myocardial ischemia injury and that the cardioprotection of M_3_-mAChR against myocardial ischemia is at least partly mediated by the downregulation of miR-376b-5p. The present observations expand our understanding of the cardioprotection of M_3_-mAChR associated with ischemic heart disease and may lead to rational target selection for therapeutic intervention.

## Supporting Information

Figure S1 **MiRNA transfection efficiency.** (A) Representative H9c2 cell photomicrographs transfected with miRNA with fluorescence. (B) MiR-376b-5p level was significantly up-regulated after transfection with miR-376b-5p. H9c2 cells were transfected with miR-376b-5p for 24 h. MiR-376b-5p level was determined by quantitative real-time RT-PCR (qRT-PCR). Note: Values are expressed as mean ± SEM; n = 3 independent experiments; *P<0.05 versus Control.
(DOC)
