# Supplementary material for: M3 Subtype of Muscarinic Acetylcholine Receptor Promotes Cardioprotection via the Suppression of miR-376b-5p
Source: PLoS One. 2012 Mar 2;7(3):e32571. doi: 10.1371/journal.pone.0032571 (PMC3292572; doi:10.1371/journal.pone.0032571)
Supplement: Figure S1 — MiRNA transfection efficiency. (A) Representative H9c2 cell photomicrographs transfected with miRNA with fluorescence. (B) MiR-376b-5p level was significantly up-regulated after transfection with miR-376b-5p. H9c2 cells were transfected with miR-376b-5p for 24 h. MiR-376b-5p level was determined by quantitative real-time RT-PCR (qRT-PCR). Note: Values are expressed as mean ± SEM; n = 3 independent experiments; *P<0.05 versus Control. (DOC) [file pone.0032571.s001.doc]

**
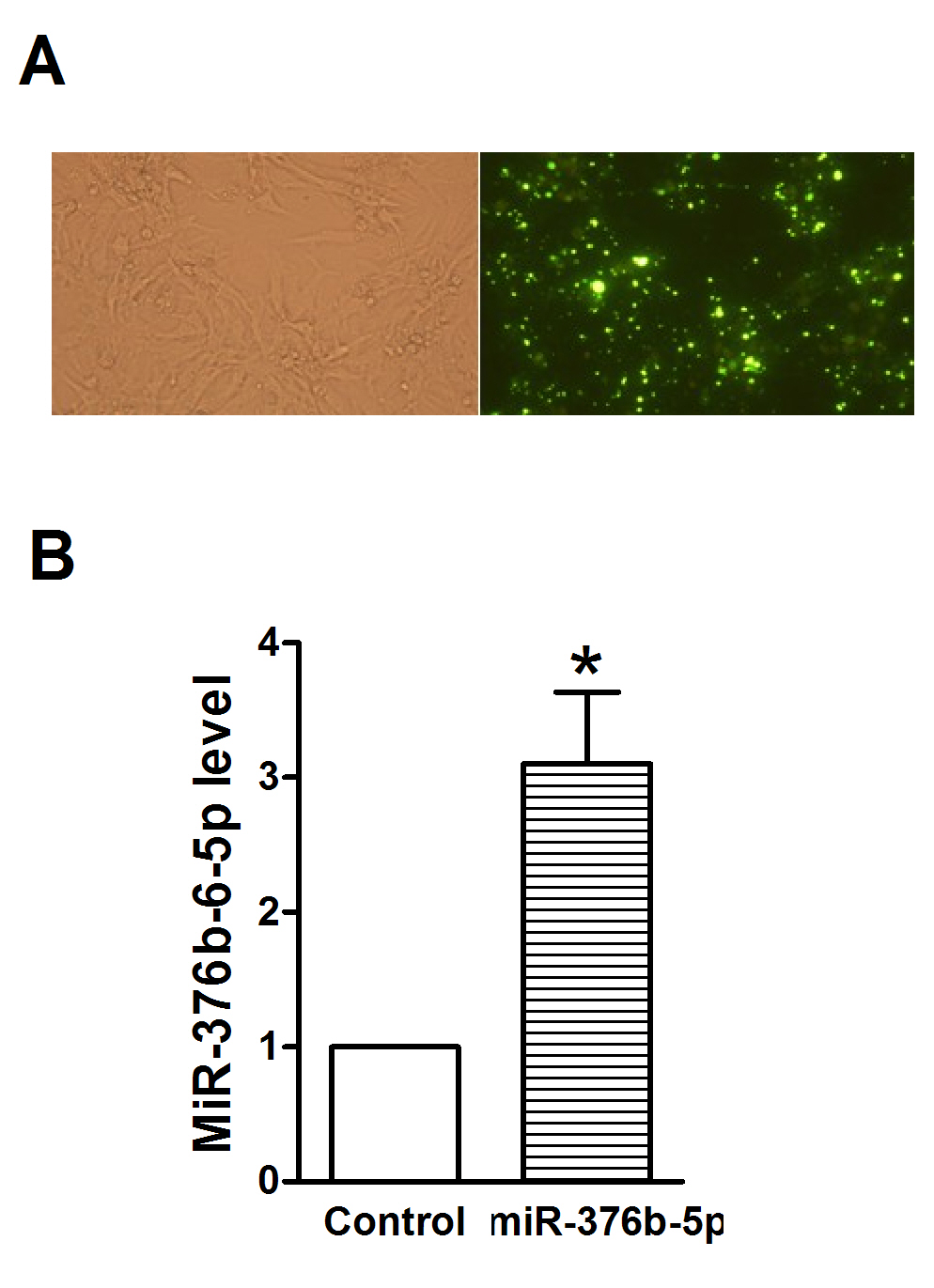
**

**Figure S1. MiRNA transfection** [**efficiency**](http://www.iciba.com/efficiency/)**.** (A) Representative H9c2 cell photomicrographs transfected with miRNA with fluorescence. (B) MiR-376b-5p level was significantly up-regulated after transfection with miR-376b-5p. H9c2 cells were transfected with miR-376b-5p for 24 h. MiR-376b-5p level was determined by quantitative real-time RT-PCR (qRT-PCR). Note: Values are expressed as mean ± SEM; n=3 independent experiments; **P* < 0.05 versus Control.
